# Effects of Erythropoietin in White Adipose Tissue and Bone Microenvironment

**DOI:** 10.3389/fcell.2020.584696

**Published:** 2020-11-24

**Authors:** Sukanya Suresh, Jeeyoung Lee, Constance Tom Noguchi

**Affiliations:** Molecular Medicine Branch, National Institute of Diabetes and Digestive and Kidney Diseases, National Institutes of Health, Bethesda, MD, United States

**Keywords:** brain, fat, macrophage, osteoclast, osteoblast, bone, erythropoietin, microglial

## Abstract

Erythropoietin (EPO) is expressed primarily in fetal liver and adult kidney to stimulate red blood cell production. Erythropoietin receptor expression is not restricted to erythroid progenitor cells, and non-erythroid EPO activity includes immune response and bone remodeling. In bone fracture models, EPO administration promotes bone formation and accelerates bone healing. In contrast, in healthy adult mice, exogenous EPO-stimulated erythropoiesis has been concomitant with bone loss, particularly at high EPO, that may be accompanied by increased osteoclast activation. Other EPO-associated responses include reduced inflammation and loss of fat mass with high-fat diet feeding, especially in male mice. While EPO exhibited a sex-dimorphic response in regulation of fat mass and inflammation in obese mice, EPO-stimulated erythropoiesis as well as EPO-associated bone loss was comparable in males and females. EPO administration in young mice and in obese mice resulted in bone loss without increasing osteoclasts, suggesting an osteoclast-independent mechanism, while loss of endogenous EPO decreased bone development and maintenance. Ossicle formation of bone marrow stromal cell transplants showed that EPO directly regulates the balance between osteogenesis and adipogenesis. Therefore, during development, endogenous EPO contributes to normal bone development and in maintaining the balance between osteogenesis and adipogenesis in bone marrow stromal cells, while EPO treatment in mice increased erythropoiesis, promoted bone loss, decreased bone marrow adipogenesis, and increased osteoclast activity. These observations in mouse models suggest that the most prevalent use of EPO to treat anemia associated with chronic kidney disease may compromise bone health and increase fracture risk, especially at a high dose.

## Introduction

Erythropoietin (EPO) regulates red blood cell production, and recombinant EPO is used primarily to treat anemia in chronic kidney disease. EPO can stimulate additional non-erythroid cell response mediated by erythropoietin receptor (EPOR) expression in other hematopoietic cells and non-hematopoietic tissues including white adipose tissue (WAT), brain, and bone (Suresh et al., 2019b). In mice, EPO treatment is associated with anti-inflammatory activity, neuroprotection, cardioprotection, skeletal muscle wound healing, protection against diet-induced obesity (Zhang et al., 2014; Suresh et al., 2019b), and bone loss accompanying EPO-stimulated erythropoiesis (Hiram-Bab et al., 2015; Suresh et al., 2019a). Hormones, cytokines, and growth factors can influence osteoblast and osteoclast differentiation, bone remodeling, and macrophage immune response, and the resultant production of inflammatory cytokines can modulate osteoclast differentiation and bone resorption activity (Sinder et al., 2015; Adamopoulos, 2018; Kubatzky et al., 2018). Here we describe the EPO response of cells derived from the monocyte–macrophage lineage such as the anti-inflammatory EPO response during diet-induced obesity in white adipose tissue and brain and the osteoclasts associated with EPO-stimulated bone loss. We also include a discussion of EPO effects on fat accumulation during diet-induced obesity and on bone marrow stromal cells associated with EPO-stimulated bone loss.

## Sex-Specific EPO Regulation and Diet-Induced Obesity

The estrogen-dependent EPO activity was first identified with EPO angiogenic response in the uterus during the mouse estrus cycle (Yasuda et al., 1998). Among non-hematopoietic tissues, EPOR has a relatively high expression in brain and adipose tissue. The EPO-stimulated hypoxic ventilatory response in brain and carotid body is also sex dimorphic and is increased in females compared with males in human and mouse (Soliz et al., 2012). With respect to adipose tissue, mice with erythroid-restricted EPOR expression show a disproportionate accumulation of fat mass that is greater in females that develop obesity and insulin resistance at a younger age compared with their male counterpart (Teng et al., 2011).

### EPO Protection in Mouse Adipose Tissue Inflammation

C57BL/6 mice fed a high-fat diet develop an obese phenotype and inflammation in WAT. With obesity, the macrophages in WAT stromal vascular fraction shift from an anti-inflammatory toward a pro-inflammatory phenotype, decrease the secretion of the anti-inflammatory cytokine IL-10 that enhances adipocyte insulin sensitivity, and increase the expression of inflammatory cytokines, TNFα, and IL-6, contributing to insulin resistance (Lumeng et al., 2007; Lauterbach and Wunderlich, 2017). The increased WAT inflammation and macrophage infiltration result in crown-like structures, which are macrophages surrounding necrotic or dying adipocytes, indicating the proinflammatory state of adipose tissue.

In mice, EPO contributes directly to metabolic homeostasis by maintenance of WAT. During high-fat-diet feeding, EPO treatment protects against glucose intolerance, insulin resistance, WAT inflammation, and fat mass accumulation, particularly in male mice (Wang et al., 2014; Zhang et al., 2014; Alnaeeli and Noguchi, 2015). In WAT, EPOR is highly expressed in adipocytes and primarily in macrophages in the stromal vascular fraction (Alnaeeli et al., 2014; Alnaeeli and Noguchi, 2015). EPO treatment significantly reduced the inflammation in WAT, macrophage infiltration, and crown-like structures and promoted an anti-inflammatory phenotype (Alnaeeli et al., 2014). EPO directly stimulated EPOR-expressing macrophages by activating STAT3, reducing proinflammatory gene expression and increasing IL-10 expression. The protective effect of endogenous EPO in metabolic control was demonstrated in mice with EPOR restricted to erythroid tissue (Suzuki et al., 2002). Subjected to high-fat-diet-induced obesity, these mice with similar body weight and fat mass as wild-type mice had greater glucose intolerance and insulin resistance, increased WAT inflammation and macrophage infiltration, enhanced crown-like structures, and increased inflammatory cytokine production (Alnaeeli et al., 2014). In the liver, EPO inhibited gluconeogenesis, attenuated obesity-related inflammatory cytokine expression and production of TNF-α and IL-6, reduced the activation of NFκB and inflammatory signaling, and enhanced insulin-related PI3K signaling (Meng et al., 2013). In the pancreas, EPO exerted JAK2-dependent protective effects in pancreatic β-cells and induced proliferative, anti-inflammatory, and angiogenic activity within the islets in diabetic mouse models (Choi et al., 2010).

The expansion of adipose tissue requires increased adipose tissue vasculature to maintain appropriate blood flow to supply oxygen and nutrients. A deficit in adipose tissue angiogenesis may contribute to insulin resistance and metabolic disease (Corvera and Gealekman, 2014). It has been suggested that vascular dysfunction resulting in adipose tissue hypoxia may lead to obesity-associated inflammation and occur before insulin resistance (Ye, 2011). Adipose tissue vasculature has been considered as a potential target for type 2 diabetes (Corvera and Gealekman, 2014). In retinoic acid treatment, activation of the brown fat-associated program in white adipocytes was mediated by vascular endothelial growth factor-stimulated angiogenesis (Wang et al., 2017). With exercise, vascularization in subcutaneous WAT is increased and may mediate an improved metabolic response (Min et al., 2019). EPO exhibits angiogenic and neovascularization activity in animal models (Yasuda et al., 1998; Ribatti et al., 1999; Kertesz et al., 2004; Wang et al., 2004; Li et al., 2007). EPO treatment in patients with Friedreich ataxia suggested the possibility for EPO to increase capillary density in skeletal muscle (Nachbauer et al., 2012). Further investigation is warranted to determine whether EPO treatment promotes vascularization, particularly in white adipose tissue, to contribute to a potentially beneficial metabolic response.

### Sex-Specific EPO Regulation of Fat Mass and Inflammation

Exogenous EPO treatment for 3 to 4 weeks in male, but not female, C57BL/6 mice reduced fat mass accumulation (Zhang et al., 2017). EPO treatment during high-fat-diet feeding in male mice increased the expression of genes PRDM16 and UCP1 associated with brown adipocyte differentiation in both brown fat and white fat (Wang et al., 2013; Kodo et al., 2017). This sex-dimorphic activity of EPO fat mass regulation is attributed to the anti-obesity effect of estrogen that interferes with EPO regulation of fat mass. In contrast to acute EPO treatment, long-term EPO treatment is able to regulate fat mass and body weight in both genders. Male and female transgenic mice with chronically increased EPO production have decreased body weight (Katz et al., 2010). Increased EPO expression by gene electrotransfer in skeletal muscle resulted in a 100-fold increase in serum EPO. By 12 weeks after the electrotransfer in female obese mice, fat mass was reduced by 28% (Hojman et al., 2009). The transfected muscle showed increased muscle volume and vascularization. However, in mice with mixed strain background, the absence of EPO receptor expression in WAT did not have any effect on adipocyte regulation (Luk et al., 2013).

The interference of estrogen with EPO regulation of fat mass was demonstrated by acute EPO treatment in ovariectomized female mice on high-fat diet that decreased fat mass accumulation, but not when combined with estradiol supplementation (Zhang et al., 2017). EPO regulation of fat mass is associated with increased activity and decreased food intake mediated by EPOR expression in non-hematopoietic tissue, especially in WAT adipocytes and in the brain (Zhang et al., 2017; Dey et al., 2020). EPOR expression in the hypothalamus arcuate nucleus localizes with proopiomelanocortin (POMC) neurons that, when stimulated, produces POMC to suppress appetite (Teng et al., 2011). EPO administration increases POMC expression in the hypothalamus and in POMC neural cell cultures (Teng et al., 2011; Dey et al., 2016). Male mice with EPOR restricted to erythroid cells exhibit increased hematocrit and reduced POMC expression, and EPO administration in these mice does not alter fat mass or POMC expression (Teng et al., 2011).

Erythropoietin is produced in the brain by astrocytes and neurons contributing to intrinsic hypoxic response (Marti et al., 1997; Bernaudin et al., 2000; Shingo et al., 2001). In animal models, a neuroprotective effect is associated with EPO activity in the brain, and EPO reduces neuronal cell death and inflammation associated with brain ischemia, with a decrease in astrocyte activation, recruitment of microglia, and proinflammatory cytokine production (Villa et al., 2003). High-fat-diet-induced obesity in mice also increases inflammation in the hypothalamus, activation of microglial cells, and inflammatory cytokine production. Hypothalamus inflammation is linked to a disruption of energy homeostasis and contributes to obesity, insulin resistance, and glucose intolerance (Jais and Bruning, 2017). Increased EPO in the brain, either by cerebral transgenic EPO expression (Tg21-mice) or by implanted EPO secreting intracerebral ventricular pump in mice, reduced fat mass accumulation during high-fat-diet feeding, reduced hypothalamus inflammation, and prevented myeloid cell recruitment to the hypothalamus in males. The unchanged hematocrit in these mice suggested an insufficient transport of EPO across the blood–brain barrier to affect EPO-stimulated erythropoiesis and showed that cerebral EPO regulation of fat mass and hypothalamus inflammation is independent of EPO-stimulated erythropoiesis (Dey et al., 2020). In contrast, deletion of *EPOR* in neural cells using the *Nestin-Cre* transgenic model showed increased weight gain, hypothalamus inflammation, and inflammatory cytokine expression in male mice, indicating that endogenous cerebral EPO also contributes to the protection against weight gain and hypothalamus inflammation during diet-induced obesity (Dey et al., 2020).

A sex-dimorphic response of cerebral EPO regulation during diet-induced obesity resulted from estrogen blocking the protective effects of EPO signaling in the brain on fat mass accumulation and hypothalamus inflammation during high-fat-diet feeding in female mice. In female ovariectomized Tg21-mice, increased EPO levels in the brain regulated body weight and hypothalamus inflammation during high-fat diet, while no such changes were evident in female non-ovariectomized Tg21-mice or in female mice with *Nestin-Cre* deletion of *EPOR* in neural cells (Dey et al., 2020). While elevated EPO improves glucose tolerance in both male and female mice, EPO regulation of fat mass accumulation and inflammation during diet-induced obesity, mediated in part *via* activity in WAT and the hypothalamus, appears to be sex specific.

## Endogenous EPO Is Required for Bone Development

Lineage tracing studies for EPOR expression using the Epo-R-Cre and yellow fluorescent protein reporter mice showed 75% labeling efficiency of Ter119+ cells. This suggested erythroid specificity for EPOR expression in the bone marrow microenvironment, with no yellow fluorescent protein expression in hematopoietic stem cells, B-lymphoid or myeloid lineages, or mesenchymal- and osteoblastic-enriched populations (Singbrant et al., 2011). However, other reports showed that bone marrow stromal cells (BMSCs) expressed *EPOR* mRNA and EPOR protein by flow cytometry and were EPO responsive in culture, resulting in induced osteoblastic differentiation with increased mineral deposition and alkaline phosphatase activity. EPO also stimulated osteoclastogenesis of preosteoclasts in culture (Shiozawa et al., 2010; Hiram-Bab et al., 2015). EPOR expression on BMSCs, osteoblasts, and osteoclasts thus adds to the increasing select non-erythroid cells that respond to EPO, mediated *via* EPOR cell surface expression (Shiozawa et al., 2010; Hiram-Bab et al., 2015; Suresh et al., 2019a, 2020b).

Mice with EPOR restricted to erythroid tissue (ΔEpoR_E_), generated by rescuing the *EPOR* knockout mouse with an erythroid *EPOR* transgene driven by the *GATA1* locus hematopoietic regulatory domain, demonstrated the requirement for EPO/EPOR signaling in normal bone development (Suresh et al., 2019a). These ΔEpoR_E_-mice lacked EPOR expression in BMSCs and osteoblasts, and in cultures, these osteoblasts had reduced differentiation and mineralization. ΔEpoR_E_-mice had reduced trabecular bone and no significant changes in cortical bone, suggesting that endogenous EPO–EPOR signaling is essential for proper bone development (Table 1). The ΔEpoR_E_-mice showed excessive marrow adiposity (Suresh et al., 2019a). Enhanced marrow adipogenesis is often observed with reduced osteogenesis, suggesting an imbalance in the differentiation of BMSCs (Muruganandan et al., 2018). Ectopic ossification assays in immunodeficient mice using wild-type BMSCs with intact EPO–EPOR signaling formed ossicles with a defined outer cortical bone, interspersed with trabeculae and marrow containing hematopoietic cells and adipocytes. ΔEpoR_E_-BMSCs formed ossicles with a cortical bone similar to control cells but with reduced trabecular bone and increased marrow adipocytes. These studies conclusively showed that endogenous EPO–EPOR signaling is essential in regulating normal differentiation and the lineage commitment of BMSCs, with a specific role in the development and the maintenance of the trabecular bone.

**TABLE 1:** Effects of erythropoietin in the bone *in vivo*.

Type	Genetic background	Bone phenotype	Mechanism	References
**Transgenic animal models of EPO signaling**				
Tg6	Overexpression of human EPO driven by PDGF-β promoter, males and females	Trabecular bone loss, thin cortical bone, decreased marrow adiposity – femora	Increased bone resorption and reduced bone formation rate	Hiram-Bab et al., 2015; Suresh et al., 2019a
ΔEpoR_E_	Epor−/− mice rescued by erythroid restricted EPOR transgene (GATA-1 locus hematopoietic regulatory domain driving mouse EPOR cDNA), males and females	Reduced trabecular bone, cortical bone unaffected, increased marrow adiposity – femora	Increased bone resorption with age; reduced osteogenic potential of osteoblasts	Suresh et al., 2019a
Mice with EPOR deletion in osteoblasts	Mice generated by crossing Osteocalcin (Bglap)-Cre mice with Epor^floxp/floxp^ mice, males and females	Reduced trabecular bone, cortical bone, and marrow adiposity unaffected – femora	No change in bone resorption; reduced osteogenic potential of osteoblasts; reduced osteocytes and increased empty lacunae	Suresh et al., 2020b
**EPO administration in healthy animals**				
Wild-type mice	C57BL6 strain, female	Trabecular bone loss – femora	Increased bone resorption	Hiram-Bab et al., 2015; Suresh et al., 2019a
Wild-type mice	C57BL6 strain, male	Trabecular bone loss – tibiae	Increase in both bone resorption and bone formation	Singbrant et al., 2011
Wild-type mice	C57BL6 strain, gender unknown	Increased bone mineral density and bone volume – vertebrae	Increased osteoblasts on bone surface	Shiozawa et al., 2010
ΔEpoR_E_	C57BL6 background, females	No changes in trabecular or cortical bone – femora	No changes in osteoblasts or osteoclasts	Suresh et al., 2019a
Mice with EPOR deletion in osteoblasts	C57BL6 background, males and females	Non-significant reduction in trabecular bone in males, intact trabecular bone in females, cortical bone unaffected in both males and females – femora	No changes in osteoblasts or osteoclasts	Suresh et al., 2020b

**FIGURE 1:**
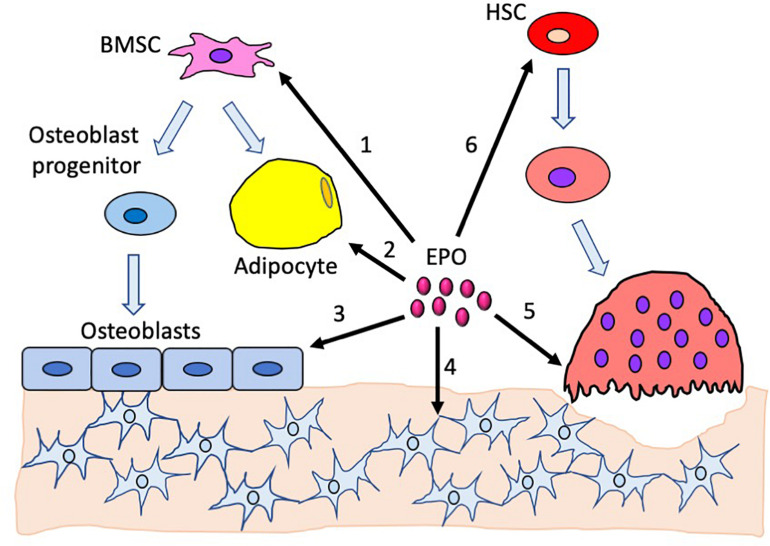
Effects of erythropoietin in the bone and the bone marrow compartment. 1. Endogenous EPO regulates bone marrow stromal cell differentiation to adipocytes and osteoblasts. 2. Increased EPO decreases bone marrow adipocytes. 3. EPO directly regulates osteoblast differentiation; the absence of EPO signaling in osteoblasts reduces their differentiation potential. Elevated EPO has a dose-dependent effect on osteoblasts, with low-dose EPO reducing differentiation and high-dose EPO increasing differentiation. 4. Absence of EPO signaling in mature osteoblasts reduces osteocytes, resulting in empty lacunae. 5. High EPO stimulates differentiation of preosteoclasts into osteoclasts. 6. EPO stimulates hematopoietic stem cells which, in turn, promotes both osteoblast and osteoclast differentiation.

Mice with *EPOR* deletion, specifically in mature osteoblasts, using the *Osteocalcin-Cre* system demonstrated the contribution of osteoblast direct response to EPO in bone development and remodeling (Suresh et al., 2020b). Both male and female mice had normal hematocrits and a significant reduction in trabecular bone volume, with the cortical bone unaffected (Table 1). The osteoprogenitors derived from calvaria had reduced differentiation and mineralization potential in cultures similar to the ΔEpoR_E_-mice. This study showed endogenous EPO–EPOR signaling in osteoblasts as an essential mechanism integral for the development of the trabecular bone (Figure 1).

### Elevated EPO in Mice Promotes Bone Remodeling Independent of EPO-Stimulated Erythropoiesis

Studies focusing on the role of EPO in bone have utilized mouse models treated with recombinant human EPO either at physiological or elevated doses for acute EPO effects or the transgenic mouse model Tg6 with constitutive overexpression of human EPO to explore the chronic effects of elevated EPO (Singbrant et al., 2011; Hiram-Bab et al., 2015; Suresh et al., 2019a). Modulation of endogenous EPO signaling in mice with Von Hippel–Lindau (VHL) mutation in osteoblasts caused an increase in hypoxia inducible factor-1/2 (HIF), resulting in elevated EPO production in osteoblasts and polycythemia (Rankin et al., 2012). In these mice, even with significant expansion of erythropoiesis, trabecular bone volume was increased. The targeted deletion of prolyl hydroxylase (PHD) in osteoblasts also induced osteoblast production of EPO and increased hematocrit, demonstrating that manipulation of the PHD/VHL/HIF pathway in osteoblasts in mice alters endogenous EPO production, elevates hematocrit, and affects bone formation. In contrast, mice with deletion of VHL, HIF-1, and HIF-2 in osteoblasts exhibited reduced EPO levels and low trabecular bone volume, with HIF-2 being the critical HIF-regulating EPO in bone (Rankin et al., 2012). In mice with PHD2 deletion in renal cells, macrophages, neural cells, and astrocytes, there was a significant reduction of bone volume due to reduced bone formation (Rauner et al., 2016). Increased EPO levels were observed in mice with PHD2 deletion, and the conditional deletion of HIF-2α in this model rescued the loss of bone. These mice exhibit increased hematocrits and elevated EPO; however, the high levels of EPO did not increase osteoclast differentiation or drive bone resorption as seen in the Tg6 mouse model, which also has chronically elevated EPO (Hiram-Bab et al., 2015). PHD2 deletion specifically in osteoblasts, however, increased bone density by reducing osteoclasts, showing that the loss of PHD2 in osteoblasts is not essential for bone reduction (Rauner et al., 2016).

The effects of elevated EPO in bone metabolism appear controversial as EPO treatment has resulted in a gain in bone density and volume in some models while reducing bone in others, suggesting a context-dependent role for EPO in bone remodeling (Hiram-Bab et al., 2017; Suresh et al., 2019b). In animal models of bone fracture, EPO treatment significantly accelerated bone healing (Holstein et al., 2007; Mihmanli et al., 2009; Rolfing et al., 2014b; Omlor et al., 2016). The process of new bone formation occurs in two ways: by endochondral ossification, where cartilage is replaced by bone tissue as seen in long bones, or by intramembranous ossification, where mesenchyme is converted to osteoblasts as seen in the formation of flat bones like the skull and the clavicle (Breeland et al., 2019). Studies done in murine fracture models showed that EPO administration promotes endochondral ossification and aids in the healing process. In cranial bone fracture models where the bone is usually repaired by intramembranous ossification, EPO administration along with bone morphogenetic protein 2 (BMP2) resulted in new bone formation by endochondral process along with increased angiogenesis possibly mediated by EPO (Sun et al., 2012). The HIF signaling pathway has also been shown to be important in EPO-stimulated repair of osteonecrosis in rat models, as EPO administration increased the expression of alkaline phosphatase, HIF-1α, runt-related transcription factor 2, and vascular endothelial growth factor (Li et al., 2018).

While EPO primarily regulates red blood cell production, most of the studies with EPO administration in healthy mouse models of different ages show a reduction in bone volume accompanying the increase in hematocrit (Singbrant et al., 2011; Hiram-Bab et al., 2015; Suresh et al., 2019a). However, in contrast to these reports, another study reported an increase in bone formation in the vertebrae of both newborn and 4–6-week-old mice receiving supraphysiological doses of EPO with a modest increase in hematocrits (Shiozawa et al., 2010; Table 1). Unlike EPO regulation of fat mass and inflammation associated with diet-induced obesity, EPO-stimulated bone loss in mice does not appear to exhibit gender bias. Chronic exposure of EPO severely disrupts bone microarchitecture as demonstrated by Tg6-mice expressing a high level of transgenic human EPO (Hiram-Bab et al., 2015). Tg6-mice show reduced trabecular bone, cortical bone mineral density, cortical bone volume (Oikonomidou et al., 2016; Suresh et al., 2019a), and thickness (Hiram-Bab et al., 2015; Table 1). Excess EPO in these mice reduces osteoblast-dependent mineral apposition and bone formation rate while simultaneously increasing the osteoclast numbers (Hiram-Bab et al., 2015). BMSCs from Tg6-mice had high EPO expression and, in ectopic ossification assays in immunodeficient mice, formed markedly reduced ossicles without a defined structure or significant bone or adipocytes (Suresh et al., 2019a). The direct effects of EPO on BMSCs were shown using culture studies, where adding EPO less than 5 U/ml to primary mouse BMSC cultures inhibited osteogenic differentiation, while elevated doses of EPO between 50 and 250 U/ml increased their osteogenic differentiation potential (Rauner et al., 2016). Osteogenic differentiation assays of BMSCs in the presence of EPO have been reported to activate EphrinB2/EphrinB4 (Li et al., 2015) mTOR (Kim et al., 2012) and JAK2/PI3K pathways (Rolfing et al., 2014a). In BMSC cultures and *in vivo*, the administration of EPO increased BMP2 expression in hematopoietic stem cells, which have been suggested to increase osteoclasts initially, followed by a rise in osteoblast numbers (Shiozawa et al., 2010). Wnt signaling has been proposed to mediate EPO effects in non-hematopoietic cells. Endothelial cell cultures exposed to elevated glucose suggested that EPO cytoprotection was mediated *via* Wnt1 and inhibition of glycogen synthase kinase activity (Chong et al., 2007). This led to the suggestion of potential application of EPO and Wnt signaling for the prevention of neurovascular injury associated with diabetes mellitus (Maiese, 2008). In human bone marrow-derived mesenchymal stem cell cultures, EPO induced neurogenic differentiation, especially at reduced oxygen, and stimulated increased Wnt3a- and Wnt3a-mediated EPO neuroprotection against glutamate toxicity (Danielyan et al., 2009). Wnt signaling was also shown to mediate the EPO-stimulated differentiation of mesenchymal stromal cells to osteoblasts. In cultures of human bone marrow mesenchymal stromal cells from young donors, EPO stimulated osteoblast-specific gene expression and osteoblast mineralization, but not in cultures from patients with myelodysplastic syndromes and old healthy donors that exhibited a reduced expression of genes associated with the canonical Wnt pathway. Activation of the Wnt pathway in these cells restored EPO-associated osteoblast differentiation (Balaian et al., 2018).

The polycythemia mouse model with JAK2 V617F mutation in hematopoietic cells gives rise to constitutively active EPOR, low EPO concentrations in the serum, low trabecular bone volume, and reduced osteoblast number (Oikonomidou et al., 2016). Thus, both low and elevated EPO or EPOR activation impaired osteoblast differentiation and function. In patients with thalassemia, low bone mass developing into osteoporosis is a common complication due to a myriad of factors like excess ineffective erythropoiesis, iron overload, or endocrine dysfunction seen during the disease (Wong et al., 2016). A mouse model of β-thalassemia major *th3* has high serum EPO levels (Rivella et al., 2003), reduced trabecular bone volume, and thin cortical bone (Vogiatzi et al., 2010). These mice provide examples of increased EPO or EPOR signaling, resulting in increased erythropoiesis and decreased bone formation or bone loss. However, while exogenous EPO administration increased hematocrit in ΔEpoR_E_-mice, there was no accompanying reduction in trabecular bone (Suresh et al., 2019a), suggesting that EPO-stimulated bone loss is mediated by non-hematopoietic response and independent of increased erythropoiesis. In female mice lacking EPOR signaling in osteoblasts, exogenous EPO administration did not reduce trabecular bone volume, while in male mice a similar treatment resulted in only a trend for reduced trabeculae (Suresh et al., 2020b; Table 1). These findings show that bone loss during EPO-stimulated erythropoiesis is mediated in part by direct EPO–EPOR signaling in osteoblasts.

### EPO-Mediated Bone Remodeling *Via* Osteoclast-Dependent Mechanisms

In cocultures of bone marrow monocytes and calvarial osteoblasts, addition of both EPO and erythroblasts did not stimulate osteoclast differentiation (Singbrant et al., 2011). However, in bone marrow-derived cultures, EPO did not affect preosteoclast proliferation but increased osteoclast differentiation *via* JAK2 and PI3K pathways (Hiram-Bab et al., 2015). EPO stimulated osteoclastogenesis in culture even at a low dose (Hiram-Bab et al., 2017). Other studies using bone marrow-derived cultures showed that EPO increased the osteoclast numbers, but not activity (Shiozawa et al., 2010). An osteoclast differentiation assay of RAW264.7 mouse monocyte/macrophage cell line similarly shows that EPO increased the osteoclast numbers, but not osteoclast activity (Li et al., 2015). It was suggested that EPO contributes to the communication between differentiation of osteoclasts and osteoblasts through the EphrinB2/EphrinB4 signaling pathway and increases the number of EphrinB2-expressing osteoclasts and EphrinB4 expression in ST2 stromal cells to promote osteoblastic differentiation that may play a role in bone formation (Li et al., 2015). EPO-stimulated osteoclastogenesis in bone marrow mononuclear cell cultures and RAW264.7 cells was mTOR signaling dependent, but the increased expression of the master transcription regulator of osteoclast differentiation NFATC1 in EPO-treated osteoclasts was independent of mTOR signaling in RAW264.7 cells (Kim et al., 2012; Kim and Kim, 2014).

Bone loss with elevated EPO has been associated with EPO-stimulated osteoclastogenesis (Hiram-Bab et al., 2015). Tg6-mice with elevated EPO have an increased bone marrow preosteoclast number, with 25% more osteoclasts per bone surface, and increased serum levels of bone resorption marker TRAP5b. Increased preosteoclast and osteoclast numbers and TRAP5b serum levels were also observed with EPO treatment in wild-type mice (Singbrant et al., 2011; Hiram-Bab et al., 2015). The increase in osteoclasts in mice with EPO stimulation has been reported to be dependent on the duration of EPO exposure, as a 2-week EPO administration increased the osteoclasts which appeared to decline with two more weeks of EPO treatment (Shiozawa et al., 2010). HIF signaling has also been shown to be important in osteoclast function. Inactivation of PHD in osteoblasts, leading to the specific activation of HIF-1α, has been associated with an increased production of osteoprotegerin, which blocks RANKL and RANK interaction to limit osteoclast differentiation (Shao et al., 2015), and increased interleukin-33 to reduce bone marrow-derived monocyte osteoclastic differentiation (Kang et al., 2017). Since PHD inactivation in osteoblasts increased HIF-2α and EPO (Rankin et al., 2012), these observations suggest that hypoxia induction of EPO may also restrict the expansion of osteoclastogenesis in support of other osteoclast-independent mechanisms contributing to EPO-stimulated bone loss.

### EPO-Mediated Bone Loss *Via* Osteoclast-Independent Mechanisms

Studies on the role of EPO signaling in osteoclasts showed elevated EPO directly stimulating osteoclasts in C57BL6/J mice treated with EPO and in Tg6-mice with chronically elevated EPO resulting in bone loss (Hiram-Bab et al., 2015). However, other studies observed reduction in bone volume but without an increase in osteoclast activity in C57BL/6 mice given exogenous EPO (Suresh et al., 2019a) or in C57BL/6 mice transplanted with bone marrow from Tg6-mice to induce polycythemia (Oikonomidou et al., 2016). The polycythemia JAK2^V617F^ mouse model with constitutively active EPOR has reduced bone and normal osteoclast numbers (Oikonomidou et al., 2016). β-Thalassemia major *th3* mouse model with high serum EPO and reduced bone exhibits decreased bone formation and resorption, suggesting overall reduction in bone remodeling (Rivella et al., 2003). The reduced osteoclast numbers in these mice show that elevated EPO stimulating osteoclasts is not always required for bone loss.

ΔEpoR_E_-mice were developed for erythroid-restricted expression of EPOR (Suzuki et al., 2002), but the presence of *GATA1* hematopoietic regulatory domain in the preosteoclasts results in EPOR expression in these cells (Suresh et al., 2019a). The presence of EPO–EPOR signaling in osteoclasts and its absence in other key bone regulatory cells like BMSCs and osteoblasts led to some interesting observations of endogenous EPO signaling in osteoclasts depending on the age of the mice. As ΔEpoR_E_-mice age, accumulation of excess fat mass due to the absence of endogenous EPO signaling in WAT results in increased systemic inflammation (Teng et al., 2011). The body weight of young ΔEpoR_E_-mice (8 weeks) was comparable to that of wild-type mice, and these young ΔEpoR_E_-mice did not have any increase in osteoclasts on their bone surface. However, at this age, these mice have a reduced trabecular number with increased spacing, indicating an early disruption of trabecular microarchitecture, but bone mineral density and volume were unaffected (Suresh et al., 2019a). By 11 weeks of age, ΔEpoR_E_-mice gain substantial body weight due to increased body fat mass and have about 40% reduction in trabecular bone volume and increased osteoclasts, in both male and female mice. Thus, in young ΔEpoR_E_-mice, reduction in bone is an osteoclast-independent mechanism. This study shows that dysregulation of WAT stemming from lack of EPO signaling results in inflammation and increased osteoclastogenesis with age, which adversely affects the bone.

### EPO Administration and Bone Remodeling in Mice on High-Fat Diet

In mice, the effects of EPO signaling in the bone have been largely studied in the trabecular bone compartment which comprises 20% of bone mass and is interspersed with bone marrow and adipocytes, with a higher remodeling rate than the cortical bone (Singbrant et al., 2011; Hiram-Bab et al., 2015; Suresh et al., 2019a). The cortical bone is stiffer but brittle and is essential for maintaining structure and mechanical loading (Ott, 2018). In C57BL/6J mice fed a high-fat diet, EPO administration showed distinct effects in trabecular and cortical bone compartments (Suresh et al., 2020a). On regular chow diet, daily EPO administration for 10 days reduced the trabecular bone and the cortical bone volumes, with a reduction of cortical bone osteocytes and periosteal osteoblasts surrounding the cortical bone (Suresh et al., 2019a). In contrast, the cortical bone in mice on high-fat diet was unaffected by EPO administration, but excessive marrow fat accumulation in these mice was completely abrogated by EPO administration. High-fat-diet feeding increased the osteoclast numbers but was not further increased with EPO treatment (Suresh et al., 2020a). Therefore, EPO-induced reduction of bone can affect various types of cells in the bone and marrow compartment under different dietary conditions.

## Conclusion

Studies in animal models show that, beyond stimulating erythropoiesis, EPO can also regulate fat mass and monocyte–macrophage-derived cell response in obesity-associated inflammation and bone remodeling. The potential for EPO to regulate fat mass and the increase in EPO production at high altitude may contribute to the lower obesity rate observed in humans residing at a high altitude (Voss et al., 2014; Diaz-Gutierrez et al., 2016). Sex-dimorphic EPO response is related to estrogen in female mice, which interferes with EPO protective activity in fat mass regulation and obesity-related inflammation. The relationship between EPO and body weight in humans is suggested in a subset analysis of full-heritage Pima Indians with a high prevalence of obesity and type 2 diabetes. This study showed that endogenous plasma EPO negatively associated with percent weight change per year in males, while the females exhibited a positive association (Reinhardt et al., 2016). In the bone, EPO activity is mediated *via* osteoclast-dependent and osteoclast-independent mechanisms and can affect bone healing or stimulate bone loss with increased erythropoiesis (Figure 1). Wild-type mice with EPO administration exhibit both increased erythropoiesis and bone loss, while mouse models with EPOR deletion, either in non-hematopoietic cells or specifically in osteoblasts, have increased erythropoiesis but no accompanying bone loss. Consistent with EPO promoting bone healing in animal models of fracture, a pilot study in 60 patients with tibiofibular factures who received either EPO or saline showed patients in the EPO receiving arm having faster union rates by a couple of weeks and reduced nonunion fracture (Bakhshi et al., 2013). However, in a recent study, high EPO level was also associated with higher fracture risk independent of hemoglobin and age in elderly Swedish men (Kristjansdottir et al., 2020), suggesting that, as observed in mice, EPO may also affect bone homeostasis, and chronic EPO treatment may impact on bone health. EPO stimulated FGF23 production in mouse and human, increasing serum FGF23 and reducing serum phosphate, and may contribute to elevated FGF23 in chronic kidney disease patients receiving EPO (Clinkenbeard et al., 2017). Increased FGF23 in response to EPO administration has been suggested as a possible mechanism of EPO-induced bone reduction associated with disrupted mineralization (Clinkenbeard et al., 2017). EPOR expression in non-erythroid tissues, including BMSCs, adipocytes, osteoblasts and osteoclasts, contributes to EPO activity independent of erythropoiesis that regulates bone formation and bone marrow microenvironment (Suresh et al., 2019a, 2020b). These activities demonstrated in animal models suggest that, while some non-hematopoietic EPO responses may be protective, bone health may be adversely affected with chronic EPO treatment such as that associated with chronic kidney disease.

## Author Contributions

SS, JL, and CN contributed to the planning, writing, and editing of this manuscript. All authors contributed to the article and approved the submitted version.

## Conflict of Interest

The authors declare that the research was conducted in the absence of any commercial or financial relationships that could be construed as a potential conflict of interest.
